# The Dental Student

**Published:** 1895-10

**Authors:** William W. Belcher

**Affiliations:** Seneca Falls, N. Y.


### THE DENTAL STUDENT.

BY WILLIAM W. BELCHER, D.D.S., SENECA FALLS, N. Y.

"She taught the child to read, and taught so well,
That she herself by teaching learned to spell."—BYRON.

No subject in dentistry should command more of our careful attention than that of the student. Some of us there are who refuse to discuss the student as a personal matter, a living, burning issue. Away with him, ye youth of inexperience!

That this is a condition and not a theory that confronts us was amply illustrated in the report of the Secretary of the New York State Dental Society on the enforcement of the law of 1892, requiring every person engaging in the study of dentistry to file with the Board of Censors a certificate of studentship. The Secretary in his report says,[1] "This portion of the law has been of great value in three things: First, in revealing the large number of persons engaged in dentistry claiming to have been students ten, twenty, thirty, and two or three even thirty-five years. Secondly, the enormous percentage in the first district,—sixty-four per cent. of those registering coming from New York City. Thirdly, *the small percentage of those claiming to be pupils of recognized reputable practitioners of our profession.* Out of the one hundred and forty-two students that have actually registered, *eleven* only have preceptors that are members of either the State or district societies,—twelve and one half per cent., and if we were to take the three hundred and seventy-two asking for blanks, the percentage would be still less."

This statement of facts is, to say the least, startling. Only twelve and one-half per cent. of the students of the State of New York are with members of our State and district societies!

It is not claimed that all the worthy members of our profession are society members, but it none the less goes to prove that a large percentage of our students of to-day are either content or compelled to take up the study of dentistry with the "dental associations" and "dental parlors." It also goes to prove that our society members are not doing their duty in this important work, leaving the field to be tilled by the less worthy members of our profession; for it is

---

[1] New York State Dental Society Transactions, 1893, p. 12.

a fact that the members of our dental societies are the representative men in their section.

Granting the teaching of the "dental parlors" to be *par excellence*, what can you say of the ethics? Association is everything; "like father, like son," is the old saying. When the student has been taught to look at the society with suspicion, that fifty cents is the correct price for filling teeth,—more is extortion and robbery,—that advertising "pays," and a cheap article of dentistry is the proper thing, what can you expect of the future?

"Not for thousands of dollars would I have a student in my office," says one prominent member of our profession. Here we have the trouble. Talk as you may of "legislation" and the "elevation of the college,"—legislation may suppress total ignorance and even empiricism to a degree, but it cannot force mediocrity into thorough competence. The colleges have done much; they can only lead the way; nothing can take the place of thorough office instruction, not only in operative technics but in professional ethics and business methods. The advancement of the profession is in our own hands; to cleanse the stream we must purify the source; to the student we must look for the future dignity and usefulness of our profession,—its further advancement and uplifting. How careful, then, should be the selection of the student! It is unnecessary to say that among the first prerequisites should be sufficient mechanical ability, education, and refinement.

He should be of good morals and character; the public judge you by your students, by the raw material, therefore let the standard be high, as a student of low morals or character cannot help but reflect on your office and yourself.

Take a personal interest in your student,—you will both learn; to my mind it has ever been a question who learned the most, the student or myself. How easy it is to forget! What perplexing questions! but you are expected to know; and it is rather humiliating to confess ignorance of a subject with which you have been and ought now to be familiar; therefore the student is a stimulant, an incentive to review work long neglected and forgotten.

Don't expect impossibilities of the boy; do you not see your own mistakes reviewed before you? It is given to only a few of us to be "born dentists." Save and protect us from some of the specimens we have encountered!

Do not make a playfellow of him; it may be that no man yet was ever a hero in the eyes of his valet, but let that be no excuse for sacrificing your dignity or authority. Keep him in the laboratory; he will have plenty of opportunities at college to learn operating; the country is full of second-class operators, but good mechanical workmen are like angels' visits,—few and far between.

Teach him that he must first acquire principles before he can apply them, that the study of physiology is impossible without a thorough knowledge of anatomy.

See to it that he selects a first-class school for his education,—*you* are responsible for this. You may say that it makes no difference so long as he has a diploma. No, indeed! there are colleges and colleges, diplomas and diplomas. The public are becoming rapidly educated; they know the good and the poor schools. Talk with some of your patients and you will be surprised at their knowledge. Graduation from a first-class college is a benefit all through life; one makes associates and acquaintances that are of incalculable value; the better the school the more desirable the associates.

He comes back from college, "simple as a child, green as a salad," to tell you "the latest." How boldly he offers suggestions, ay, even criticism! Deal with him kindly; he will soon see the error of his ways. He is in his own mind a greater man, a better dentist, than he ever will be in the future; disappointments, failures, trials, temptations, and the loss of self-confidence will insure a balance,—they come to all of us. What one finds the world just as he likes?

Youth and new wine each need the mellowing influence of time to bring them to their greatest perfection. Wine is wine, the juice of the grape. Yet time and age do not necessarily bring to it excellence; care, experience, and attention are necessary in its production; so in youth, golden opportunities and natural ability are not always the forerunners of prosperity and success,—age, experience, adversity, temptations resisted, hopes deferred, trophies unwon, all go to the formation of character, life, the crucible; cares, the fiery furnace, freeing from dross, forming true character and manhood.

Give him good advice and, best of all, good example,—not that he needs the advice or will accept it.

Tell him of your mistakes; not that he ever expects to make any, oh, dear, no! It is surprising how little we learn from others; their failures are nothing to us; we are so constituted that we cannot to any degree profit by another's experience. If we only could begin where another man left off; if he might endow us with his knowledge and experience the same as his goods and chattels, how wise we might be!

It has been truthfully said, "There is no royal road to learning;" every man must have the experience of the man before him; after a time comes judgment, ay, even conservatism, if you please.

Time passes, and our young man starts out for himself, only to find that a college education and high-class standing are not always the open sesame to practice. The practical application of principles is not so easy, but, like Antæus of old, rising from each defeat with added strength, the elusive god of prosperity smiles on him. Really, the boy is doing better than yourself. Are you jealous? Not a bit; he is yours,—your creation; you glory in his success and exult in his prosperity. After a time it is supposed you go to see him; how proud he is of his office, of his acquaintances, and the boy actually seems to be proud of you! How prosperous everything appears! Let us hope it is not all on the surface; that he is an exception, a true *rara avis*,—a dentist and a business man.

It is to be hoped he will continue to be a student, to learn what little he may of the boundless store of knowledge; that he will attend the dental meetings, exchange ideas with his brethren, and be superior to the little jealousies which are always marks of the narrow mind, and become the master-craftsman, looking down the vista of time to the twenty triumphs behind him, recalling with pleasure the paths which have led him to fame, and at last, like unto the Moses of old, behold the promised land denied to him, but of which he and his time have been the auspicious pledge,—a professional standing, yet higher, greater, and better, in the age yet to come.

